# Biosynthesis of Gold Nanoparticles by Vascular Cells *in vitro*

**DOI:** 10.3389/fmicb.2022.813511

**Published:** 2022-04-11

**Authors:** Michael Kitching, Saikumar Inguva, Meghana Ramani, Yina Gao, Enrico Marsili, Paul Cahill

**Affiliations:** ^1^Department of Oral Immunology and Infectious Diseases, School of Dentistry, University of Louisville, Louisville, KY, United States; ^2^Vascular Biology and Therapeutics Laboratory, School of Biotechnology, Dublin City University, Dublin, Ireland; ^3^School of Physics, Dublin City University, Dublin, Ireland; ^4^Nanotechnology Innovation Center of Kansas State, Department of Radiation Oncology, Wayne State University, Detroit, MI, United States; ^5^Materials and Surface Science Institute, University of Limerick, Limerick, Ireland; ^6^Department of Chemical and Materials Engineering, School of Engineering and Digital Sciences, Nazarbayev University, Nur-Sultan, Kazakhstan

**Keywords:** gold nanoparticles, biosynthesis, vascular cell, *in vitro*, biogenic nanoparticles

## Abstract

Biosynthesis of gold nanoparticles (AuNPs) for antimicrobial and chemotherapeutic applications is a well-established process in microbial hosts such as bacterial, fungi, and plants. However, reports on AuNPs biosynthesis in mammalian cells are scarce. In this study, bovine aortic endothelial cells (BAECs) and bovine aortic smooth muscle cells (BASMCs) were examined for their ability to synthesize AuNPs *in vitro*. Cell culture conditions such as buffer selection, serum concentration, and HAuCl_4_ concentration were optimized before the biosynthesized AuNPs were characterized through visible spectrometry, transmission electron microscopy, X-ray diffraction, and Fourier transform infrared (FTIR) spectroscopy. BAECs and BASMC produced small, spherical AuNPs that are semi-crystalline with a similar diameter (23 ± 2 nm and 23 ± 4 nm). Hydrogen peroxide pretreatment increased AuNPs synthesis, suggesting that antioxidant enzymes may reduce Au^3+^ ions as seen in microbial cells. However, buthionine sulfoximine inhibition of glutathione synthesis, a key regulator of oxidative stress, failed to affect AuNPs generation. Taken together, these results show that under the right synthesis conditions, non-tumor cell lines can produce detectable concentrations of AuNPs *in vitro*.

## Introduction

The biological synthesis of gold nanoparticles (AuNPs) is well characterized using microbial and plant hosts for catalysis, drug delivery, therapeutics, and environmental remediation (Mishra et al., 2011; Kitching et al., 2015; Seo et al., 2015; Patil and Chandrasekaran, 2020; Kapoor et al., 2021). However, the process of AuNPs synthesis is poorly understood in mammalian cell hosts. The process of AuNPs synthesis in microbial hosts occurs in three steps: (1) bioaccumulation/sorption of metal ions, (2) metal ion reduction, and (3) capping of the growing nanoparticles. First, the precursor Au^3+^ accumulates either within the cytosol or periplasm or is passively absorbed on the cell surface (Kashefi et al., 2001; Reith et al., 2009; Das et al., 2012b). Metal ions induce oxidative stress within the cell, where the balance between reactive oxygen species (ROS) and anti-oxidants is disrupted in favor of ROS (Burtenshaw et al., 2019). ROS are a class of highly reactive molecules derived from O_2_ metabolism, which include superoxide (O_2_^–^), alkoxyl radical (RO⋅), peroxyl radical (ROO⋅), hydroxyl radicals (OH⋅), peroxynitrite (ONOO^–^), hydrogen peroxide (H_2_O_2_), ozone (O_3_), and hypochlorous acid (HOCl) (Phaniendra et al., 2015). For example, Au^3+^ ions induce oxidative stress in *Escherichia coli via* the generation of superoxide ions (O_2_^–^) (Muñoz-Villagrán et al., 2020). Bioreduction of metal ions such as Au^3+^, Cr^6+^, Pb^2+^, and Ag^1+^ is a defense response of microbial cells to the oxidative stress induced by these metal ions in solution (Fredrickson et al., 2008; Reith et al., 2009; Italiano et al., 2012). Microbial cells respond to oxidative stress induced by Au^3+^ by upregulating the expression of antioxidant enzymes such as NADH reductases (Das et al., 2012a), which reduce Au^3+^ to insoluble Au^0^ and result in Au^0^ nucleation and growth of AuNPs (Muñoz-Villagrán et al., 2020). Fungal cell catalysts such as *Rhizopus oryzae* reduce Au^3+^ to Au^0^ *via* an Au^1+^-S intermediate step, which decreases Au microbial toxicity (Reith et al., 2009; Das et al., 2012b). In the final step of AuNPs biosynthesis, the growth of the AuNPs is capped mainly by proteins derived from host cells (Das et al., 2012b). AuNPs produced by microbial cells are generally small spheres with a broad size distribution due to the poor specificity of the capping agents (Kitching et al., 2015). Further, particle size distribution is affected by the biomass: Au precursor ratio (Das et al., 2012a), synthesis temperature (Timoszyk et al., 2017), and protein extraction method utilized (Kitching et al., 2016). These parameters affect the nucleation of AuNPs and the stability of the reducing enzymes and capping proteins.

Although limited information is available on mammalian cell generation of AuNPs, Au^3+^ ions generate excess intracellular ROS in mammalian cell lines such as THP-1 (Lewis et al., 2005). At physiological concentration, ROS play an important role in intracellular communication in mammalian cells (Ushio-Fukai, 2009; Duhé, 2013; Son et al., 2013; Caliceti et al., 2014) and their production is stimulated by growth factors (Bäumer et al., 2008; Lien et al., 2014), cytokines (Weaver et al., 2012), hypoxia (Rathore et al., 2008), shear stress (Chiu et al., 1997), and cyclic strain (Cheng et al., 1998). However, excess ROS can also be generated from exogenous sources such as UV light, air and water pollution, alcohol, tobacco smoke, industrial solvents, pesticides, high temperature, and transition metals (Liochev and Fridovich, 1999). Excess of ROS results in oxidative stress, which can cause damage to cellular organelles/structures and contribute to the progression of diseases including; cancer (Marumo et al., 1997), diabetes (Sturrock et al., 2006), neurodegenerative disorders (Hwang et al., 2003), and cardiovascular disease (Liang et al., 2019). As a result, mammalian cells respond to oxidative stress *via* the production and/or activation of antioxidant enzymes such as superoxide dismutase (SOD), catalase, heme-oxygenase-1 (HO-1), NADPH quinone reductase, and γ-glutamylcysteine reductase (Juarez et al., 2008), which neutralize free radicals.

Oxidative stress-induced by metal ions can occur in vascular cells through the overproduction of ROS such as O_2_^–^ and H_2_O_2_, catalyzed by NADPH oxidase (NOX) and/or nitric oxide synthase (NOS). Pb increases intracellular O_2_^–^ and H_2_O_2_ in human coronary artery endothelial cells (HCAEC) and human vascular smooth muscle cells (HVSMC) *via* the upregulation of gp91phox, a heme-binding subunit of NOX (Ni et al., 2004). Porcine aortic endothelial cells (PAEC) also increase O_2_^–^ production *via* NOS and NOX (Coyle and Kader, 2007). These cells respond to oxidative stress by up-regulating antioxidant enzymes such as SOD, HO-1, catalase, and increasing glutathione activity (GSH). HCAEC and HVSMC respond to oxidative stress by increasing the expression of the antioxidant enzyme SOD (Ni et al., 2004). Rat cerebral-microvascular endothelial cells (RCEC) upon exposure to mild oxidative stress (10:20 μM FeCl_3_/8-HQ) increases the expression of the antioxidant enzymes manganese superoxide dismutase and HO-1 (Méthy et al., 2004). Human dermal microvascular endothelial cells (HDMEC) respond to oxidative stress induced by H_2_O_2_ by upregulating SOD, catalase, and GSH activity (Tsaryk et al., 2007).

There are no studies published to date that use vascular cells to facilitate the synthesis of AuNPs. However, other mammalian cell lines have been investigated for their ability to produce AuNPs. Tumor cell lines have been hypothesized to have a greater capacity for AuNPs synthesis, as they spontaneously produce a high intracellular concentration of ROS and reactive nitrogen species (RNS) (Wang et al., 2013). Damaging ROS must be neutralized by antioxidant enzymes to prevent cell death. Therefore, cancer cell lines may possess endogenously higher antioxidant enzymes levels than non-tumor cell lines. While the concentration of GSH related enzymes is higher in cancer tissue than the adjacent benign tissue, the concentration of other antioxidant enzymes such as SOD, MnSOD, and catalase are lower in tumor tissue (Oberley and Oberley, 1997; Zalewska-Ziob et al., 2019). Furthermore, results from studies that compare the generation of AuNP’s in non-tumor cell lines and tumor cell lines are conflicting. The tumor cell lines HepG2 (human hepatocarcinoma) and K562 (leukemia) produced a greater abundance of AuNPs upon exposure to Au^3+^ compared to the non-tumor control cell line L02 (human embryo liver cell strand), which failed to synthesize any AuNPs (Wang et al., 2013). However, the non-tumor cell line HEK-293 produced a greater abundance of AuNPs than the cervical epithelial cancer cell lines HeLa and SiHA as well as the neo-blastoma cell line SKNSH upon exposure to AuNPs (Anshup et al., 2005). These results suggest that the non-tumor cell synthesis of AuNPs is highly dependent on synthesis conditions.

We hypothesize that vascular cells exposed to HAuCl_4_ in solution will reduce Au^3+^ to Au^0^, resulting in Au precipitation and AuNPs generation. The aims of this study are: (i) to demonstrate for the first time that the vascular cell lines bovine aortic endothelial cells (BAEC) and bovine aortic smooth muscle cells (BASMC), are capable of synthesizing AuNPs, (ii) optimize the cell culture conditions for AuNPs synthesis, and (iii) discuss a possible mechanism for *in vitro* biosynthesis of AuNPs by vascular cells.

## Materials and Methods

### Reagents

Hydrogen tetrachloroaurate trihydrate (HAuCl_4_) was purchased from Fisher Scientific. Calcium chloride (CaCl_2_) and sodium chloride (NaCl) were purchased from Merck Millipore. MgSO_4_ was purchased from BDH Chemicals. All cell culture reagents were purchased from Sigma-Aldrich, including fetal bovine serum (FBS), penicillin/streptomycin cocktail, 10× trypsin-ETDA, and PBS tablets.

### Cell Culture

Bovine aortic endothelial cells and bovine aortic smooth muscle cells were sourced from the National Institute of Health (NIH) repository and maintained at 37°C and 5/95% CO_2_/air (v/v). Cell lines were cultured in RPMI 1640 media supplemented with 10% v/v foetal bovine serum (FBS) and 1% v/v penicillin/streptomycin (P/S) antibiotic cocktail. This medium formulation will be referred to as RPMI 1640 media.

### Optimization of Cell Culture Conditions for Gold Nanoparticles Biosynthesis

For AuNPs biosynthesis experiments, BAECs and BASMCs were seeded into the six-well plate at a density of 1 × 10^5^ cells per well. Confluent cell lines were washed gently with 10 mM PBS thrice immediately before AuNPs biosynthesis experiments to remove possible residual reducing agents that are present in the cell culture media.

#### Synthesis Buffer Selection

Confluent BAECs in a 6-well tissue culture plate were exposed to either 2 mL of Dulbecco’s Modified Eagle Medium (DMEM) without phenol red or 10 mM PBS containing 0, 0.5, 0.75, 1, 1.5 or 2 mM HAuCl_4_, supplemented with 1% v/v FBS. FBS concentration was initially kept low to avoid interference with the AuNPs biosynthesis process. Cells were incubated for 48 h at 37°C and 5% CO_2_.

#### Fetal Bovine Serum Optimization

Fetal bovine serum promotes cell metabolism, which may facilitate Au^3+^ bioreduction. However, FBS contains proteins that can complex metals, which reduces their bioavailability. Therefore, to maximize AuNPs generation, the concentration of FBS must be optimized. To determine the optimal FBS concentration, 2 mL of a 10 mM solution containing 0.75 mM HAuCl_4_ was supplemented with 0, 0.5, 1, 1.5 or 2% v/v FBS were added to confluent BAECs and BASMCs in a 6-well tissue culture plate. Cells were then incubated for 48 h at 37°C and 5% CO_2_.

#### Au Dose-Response

To optimize the initial concentration of Au^3+^, 2 mL of a 10 mM PBS solution containing 0, 0.5, 0.75, 1, 1.5 or 2 mM HAuCl_4_ supplemented with 1% v/v FBS were added to confluent BASMCs and BAECs in a 6-well tissue culture plate. Cells were incubated for 48 h at 37°C and 5% CO_2_.

#### Ca and Mg Ion Addition

To investigate the benefit of Ca^2+^ and Mg^2+^ ion supplementation, confluent BAECs and BASMCs in a 6-well tissue culture plate were exposed to 0, 1.5 or 2 mM HAuCl_4_ in 10 mM PBS containing 1% v/v FBS with or without 1 mM CaCl_2_ and 1 mM MgSO_4_. Cells were incubated for 48 h at 37°C and 5% CO_2_.

### Preliminary Mechanistic Study

#### Excess Reactive Oxygen Species Generation

To investigate the role of ROS in AuNPs biosynthesis by vascular cells, excess ROS generation was induced in BAECs and BASMCs by pretreating each cell line with 0, 0.01, 0.05, 0.1, 0.5, 1, and 2 mM H_2_O_2_ in full RPMI 1640 media for 24 h at 37°C and 5% CO_2_. The conditioned media was aspirated, and cell lines were washed with 10 mM PBS thrice. Immediately after washing of cells, 2 mL of a 10 mM PBS solution containing 1.5 mM HAuCl_4_ and 1% v/v FBS with 1 mM CaCl_2_ and MgSO_4_ was then added to each cell line. The cells were incubated for 48 h at 37°C and 5% CO_2_.

#### Role of Glutathione

Glutathione is an important regulator of oxidative stress in the cell. To investigate the role of GSH in Au^3+^ reduction, confluent BAECs and BASMCs in a 6-well tissue culture plate were pretreated 2 mL of full RPMI-1640 media containing 0, 1, or 2 mM buthionine sulphoximine (BSO) for 16 h at 37°C and 5% CO_2_. BSO is an irreversible inhibitor of γ-glutamylcysteine synthetase and consequently lowers cellular GSH levels. The conditioned media was aspirated, and cell lines were washed with 10 mM PBS thrice. Immediately after washing of cells, 2 mL of a 10 mM PBS solution containing 1.5 mM HAuCl_4_ and 1% v/v FBS with 1 mM CaCl_2_ and MgSO_4_ was then added to each cell line. The cells were incubated for 48 h at 37°C and 5% CO_2_.

### Gold Nanoparticles Characterization

#### Isolation

To fully characterize biogenic AuNPs, AuNPs were isolated in DI water. To achieve this, detached cells and cell debris were first removed from the conditioned media by centrifugation at 5,000 rpm for 5 min at 4°C. AuNPs were isolated from the supernatant by centrifugation at 13,500 rcf for 30 min at 4°C. The AuNPs pellet was washed thrice and then suspended in DI water. AuNPs were then characterized as described in the following sections.

#### U.V. Visible Spectrophotometry

To determine the presence and relative abundance of AuNPs, ∼1.5 mL of the AuNPs suspension was analyzed on a Cary 50 spectrophotometer (Varian, United States) at a step size of 0.1 nm and a scan rate of 10 nm.s^–1^ at 25°C. AuNPs were identified by their surface plasmon resonance (SPR) band at ∼540 nm.

#### Dynamic Light Scattering

To determine the hydrodynamic size of the isolated AuNPs, 1 mL of the AuNPs suspension was analyzed in a 3 mL plastic cuvette using a Beckman Coulter Delsa Nano C Particle Analyzer.

#### High-Resolution Transmission Electron Microscopy and Selected Area Electron Diffraction

Samples were air-dried at RT on carbon film-coated copper TEM grids and examined under high-resolution cryo-TEM (HR-TEM). Selected area electron diffraction (SAED) was also performed using the same HR-TEM.

#### X-Ray Diffraction

The crystal structure of the samples was determined by X-ray powder diffraction (XRD) pattern (Bruker AXS D8 advance diffractometer). Samples were prepared on silicon wafers by drop-casting the AuNPs suspension. Before this, the silicon wafers were placed in a 3:1 mixture of ammonium hydroxide (NH_4_OH) and hydrogen peroxide (H_2_O_2_) at 60°C for 15–20 min. The silicon wafers were washed with DI water and blown dry with N_2_. Approximately 0.5 mL of each of the AuNPs suspension was added dropwise to the etched silicon wafer and spun dried at 1,000 rcf.

#### Fourier Transform Infrared Spectroscopy (FTIR)

The chemical nature of the AuNPs surface was examined by FTIR spectroscopy (FTIR). The solvent was evaporated from the AuNPs sample at 50°C overnight. The dried MNP crystals were analyzed between 4,500 and 500 cm^–1^. The force gauge did not exceed 50%.

## Results

To determine the optimal synthesis buffer to study AuNPs biosynthesis by vascular cell lines, BAECs were exposed to increasing HAuCl_4_ concentrations in DMEM or 10 mM PBS. DMEM without phenol red was initially examined as a synthesis buffer as it would not interfere with the U.V visible spectrophotometric detection of AuNPs. While BAECs exposed to HAuCl_4_ in DMEM failed to show any difference in AuNPs generation vs. the abiotic DMEM control, BAECs exposed to HAuCl_4_ in 10 mM PBS (Supplementary Figure 1) produced a significant abundance of AuNPs, with no AuNPs generation detected in the corresponding 10 mM PBS abiotic control. This demonstrates that the optimal buffer to study AuNPs biosynthesis by vascular cell lines is 10 mM PBS. Further, the conditioned media containing HAuCl_4_ changed color from light gold/yellow to dark purple approximately 48 h of incubation with BAECs. Cells attached to the culture plate also had been stained with a purple/pink color around 48 h, which remained after PBS washing. This indicates that biosynthesized AuNPs are also either adhered to the cell surface, located in the cytosol, or both.

**FIGURE 1:**
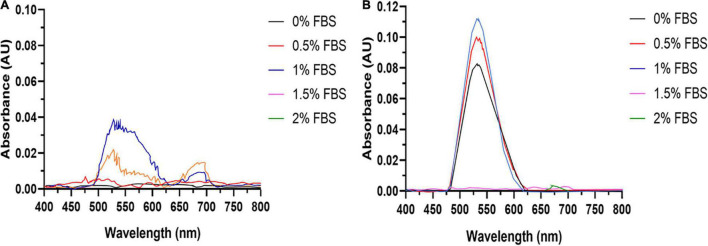
AuNPs biosynthesis is a balance between cell viability and FBS concentration. UV-Visible spectra of the AuNPs isolated from **(A)** BAECs and **(B)** BASMCs exposed to 0.75 mM HAuCl_4_ in 10 mM PBS for 48 h at 37°C (representative of *n* = 3 independent biological replicates).

Fetal bovine serum is an essential supplement in mammalian cell culture media. However, serum proteins such as albumin may complex or bind Au^3+^ ions, lowering their bioavailability. Therefore, the concentration of FBS should be minimized to increase AuNPs formation. FBS dose-response experiments reveal that the SPR peak heights for AuNPs synthesized for both cell lines are maximized at an FBS concentration of 1% v/v (Figure 1). This indicates that 1% v/v FBS is the optimal concentration for AuNPs biosynthesis by these cell lines.

**FIGURE 2:**
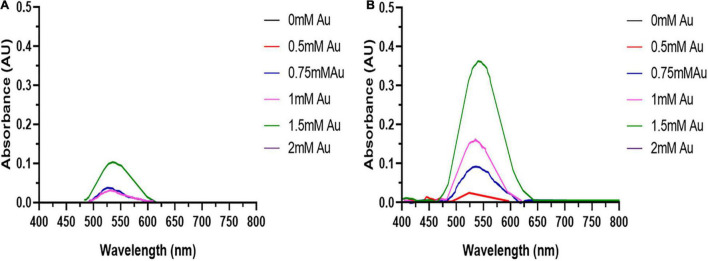
UV-visible spectra of the AuNPs isolated from **(A)** BAECs and **(B)** BASMC incubated with various concentrations of HAuCl_4_ in 10 mM PBS supplemented with 1% v/v FBS for 48 h at 37°C (representative of *n* = 3 independent biological replicates).

Due to its reactivity, Au^3+^ can bind tightly to proteins and DNA (Young Choi et al., 2013), along with the induction of oxidative stress can lead to cytotoxicity. Therefore, the optimal HAuCl_4_ concentration for AuNPs biosynthesis needs to be determined. The dose-response curves (Figure 2) show the height of the AuNPs isolated from the spent media after 48 h increases with HAuCl_4_ concentration up to 1.5 mM. Higher HAuCl_4_ concentrations result in a reduction of the SPR peak height, indicating that above a HAuCl_4_ concentration of 1.5 mM, the cytotoxicity of Au^3+^ inhibits AuNPs synthesis. Interestingly, the BASMCs appear to produce a greater abundance of AuNPs than the BAECs.

**FIGURE 3:**
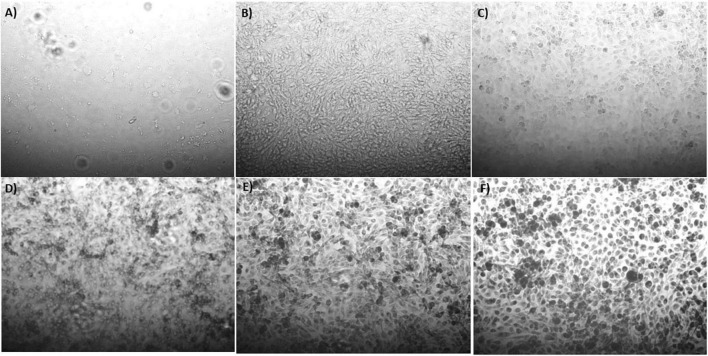
Phase contract microscope images of BAECs exposed to **(A)** 0 mM, **(B)** 0.5 mM, **(C)** 0.75 mM, **(D)** 1 mM **(E)** 1.5 mM, and **(F)** 2 mM HAuCl_4_ in 10 mM PBS and 1% v/v FBS for 48 h at 37°C. Cells were examined using phase contrast light microscopy at 100× magnification (representative of *n* = 3 independent experiments).

**FIGURE 4:**
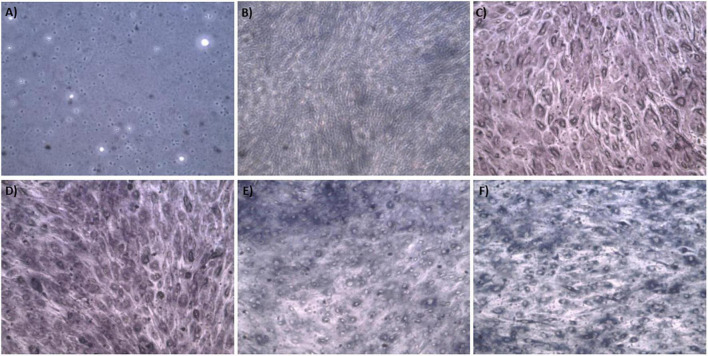
Phase contract microscope images of BASMCs exposed to **(A)** 0 mM, **(B)** 0.5 mM, **(C)** 0.75 mM, **(D)** 1 mM **(E)** 1.5 mM, and **(F)** 2 mM HAuCl_4_ in 10 mM PBS and 1% v/v FBS for 48 h at 37°C. Cells were examined using phase contrast light microscopy at 100× magnification (representative of *n* = 3 independent biological replicates).

**FIGURE 5:**
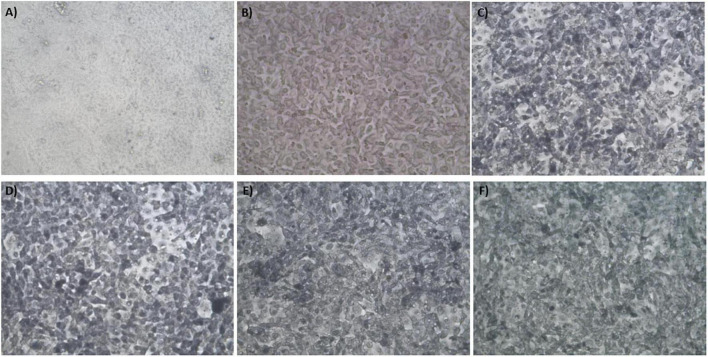
Phase contract microscope images of BAEC exposed to **(A)** 0 mM HAuCl_4_ without CaCl_2_ and MgSO_4_, **(B)** 0 mM HAuCl_4_ with 1 mM CaCl_2_ and 1 mM MgSO_4_, **(C)** 1.5 mM HAuCl_4_ without CaCl_2_ and 1 mM MgSO_4_, **(D)** 1.5 mM HAuCl_4_ with 1 mM CaCl_2_ and 1 mM MgSO_4_, **(E)** 2 mM HAuCl_4_ without CaCl_2_ and MgSO_4_, and **(F)** 2 mM HAuCl_4_ with 1 mM CaCl_2_ and 1 mM MgSO_4_ in 10 mM PBS and supplemented with 1% v/v FBS for 48 h at 37°C. Cells were examined using phase contrast light microscopy at 100× magnification (representative of *n* = 3 independent experiments).

**FIGURE 6:**
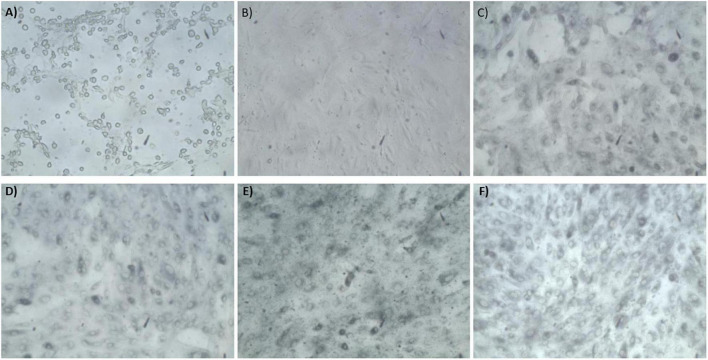
Phase contract microscope images of BASMCs exposed to **(A)** 0 mM HAuCl_4_ without CaCl_2_ and MgSO_4_, **(B)** 0 mM HAuCl_4_ with 1 mM CaCl_2_ and 1 mM MgSO_4_, **(C)** 1.5 mM HAuCl_4_ without CaCl_2_ and MgSO_4_, **(D)** 1.5 mM HAuCl_4_ with 1 mM CaCl_2_ and 1 mM MgSO_4_, **(E)** 2 mM HAuCl_4_ without CaCl_2_ and MgSO_4_, and **(F)** 2 mM HAuCl_4_ with 1 mM CaCl_2_ and 1 mM MgSO_4_ in 10 mM PBS and supplemented with 1% FBS for 48 h at 37°C. Cells were examined using phase contrast light microscopy at 100× magnification (representative of *n* = 3 independent experiments).

**FIGURE 7:**
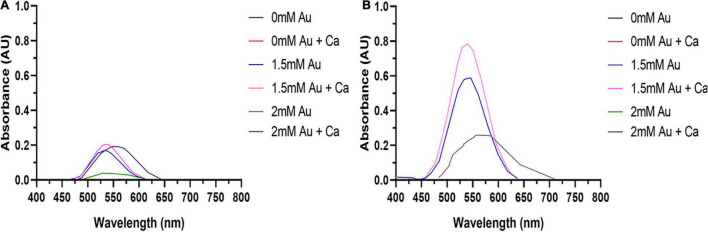
The addition of 1 mM CaCl_2_ and MgSO_4_ to **(A)** BAEC and **(B)** BASMC culture increases AuNPs synthesis. Confluent cell lines were exposed to 0, 1.5, or 2 mM of HAuCl4 with or without 1 mMCaCl_2_ and 1 mM MgSO_4_ ions for 48 h at 37°C (representative of *n* = 3 independent experiments).

The synthesis buffer chosen for AuNPs biosynthesis experiments (10 mM PBS) lacks Ca^2+^ and Mg^2+^ ions, which are important allosteric regulators of the adhesion proteins for BAEC and BASMC (Takeichi and Okada, 1972; Ko et al., 2001; Sotomayor and Schulten, 2008). The adhesion proteins facilitate cell attachment to the extra-cellular matrix *in vivo* and culture plate surface *in vitro*. Lack of these allosteric regulators results in poor cell attachment (Figures 3, 4). Interestingly, the addition of Au^3+^ ions appears to increase both BAEC and BASMC attachment. Supplementing the synthesis buffer with 1 mM MgSO_4_ and 1 mM CaCl_2_ not only resulted in better cell attachment (Figures 5, 6), but it also increases the height of the SPR peak for AuNPs produced by both BAEC and BSMC (Figure 7).

**FIGURE 8:**
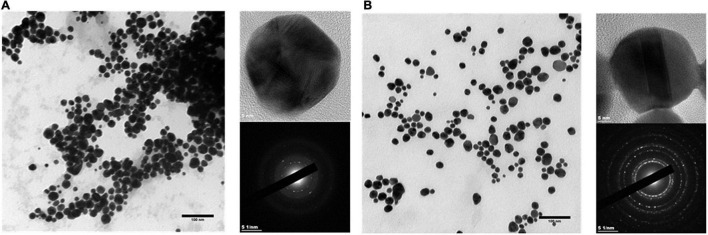
SAED and HR-TEM of AuNPs produced by **(A)** BAECs and **(B)** BASMC. Confluent cell lines were exposed to 1.5 mM HAuCl_4_ with 1 mM CaCl_2_ and 1 mM MgSO_4_ ions for 48 h at 37°C (representative of *n* = 3 independent experiments).

**FIGURE 9:**
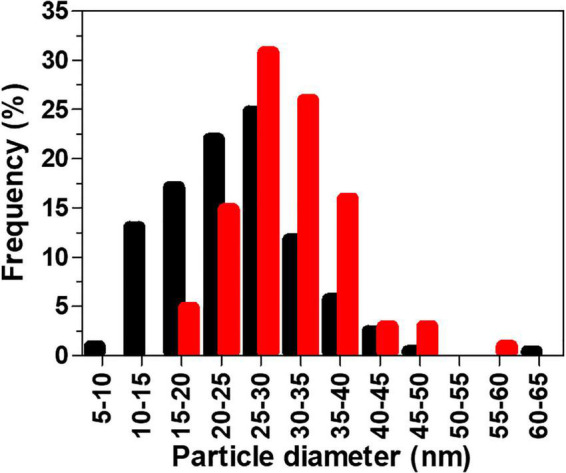
Histogram of AuNPs particle sizes (measured by TEM) produced by BAECs (black) and BASMCs (red). Confluent cell lines were exposed to 1.5 mM HAuCl_4_ supplemented with 1% v/v FBS, 1 mM CaCl_2_, and 1 mM MgSO_4_ ions for 48 h at 37°C (representative of *n* = 3 independent experiments).

AuNPs were characterized in terms of their size, shape, crystallinity, and surface chemistry. AuNPs produced by BAECs and BASMCs are mostly spherical (Figure 8). SAED shows that the AuNPs produced by the BASMCs possess more crystal faces than the BAEC synthesized AuNPs. The size distribution of the AuNPs, as determined from TEM imaging, produced by BAECs and BASMCs are slightly different. AuNPs produced by BAECs are skewed toward the smaller AuNPs size, while the BASMC produced AuNPs are skewed toward the larger AuNPs (Figure 9).

**FIGURE 10:**
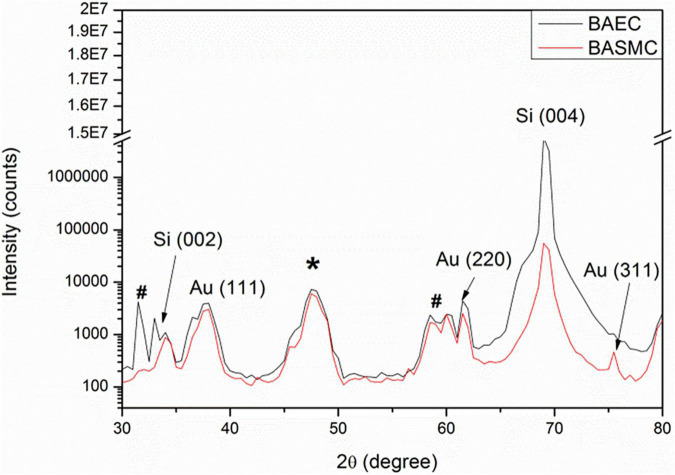
XRD of AuNPs isolated from the spent media of BAECs and BASMCs. * is due to the adhesive tape and # arises from XRD Cu Kβ and tungsten Lα radiations from the X-ray tube (representative of *n* = 3 independent experiments). Confluent cell lines were exposed to 1.5 mM HAuCl_4_ supplemented with 1% v/v FBS 1 mM CaCl_2_ and MgSO_4_ ions for 48 h at 37°C (representative of *n* = 3 independent experiments).

X-ray diffraction analysis of AuNPs produced by BAEC and BASMCs (Figure 10), showed that AuNPs produced by BAECs have Au (111) and Au (220) planes, while the AuNPs produced by BASMC have Au (111), Au (220), and Au (311) planes. The lattice spacing (d spacing) of the AuNPs was calculated using Image j analysis and used to calculate the crystal size along with the data for the Au (111) facet detected by XRD. These findings are confirmed by SAED analysis (Figure 8). Analysis of the XRD data shows that the d spacing of the AuNPs produced by both cell phenotypes is similar. However, the crystal size is much lower than the particle size calculated by HR-TEM (Table 1).

**TABLE 1:** Hydrodynamic sizes (DLS), particle size (TEM) d spacing and crystal size of the AuNPs produced by BAECs and BASMCs.

	DLS (nm)	TEM (nm)	d spacing (Å)	Crystal size (nm)
BAEC	75 ± 5	23 ± 2	2.3 ± 1	6 ± 1
BASMC	74 ± 6	23 ± 4	2.3 ± 2	5 ± 1

*P < 0.05 by Student’s t-test.*

**FIGURE 11:**
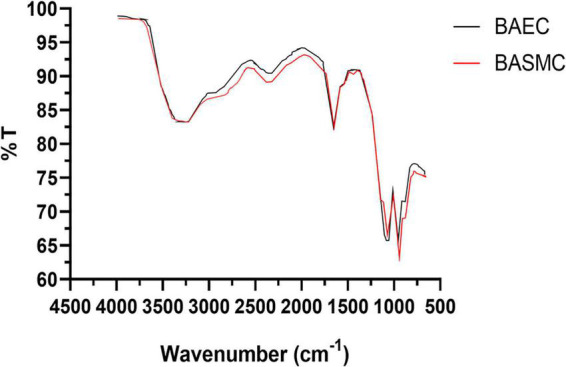
FTIR of powdered AuNPs produced by BAECs and BASMCs (representative of *n* = 3 independent experiments). Confluent cell lines were exposed to 1.5 mM HAuCl_4_ supplemented with 1% v/v FBS 1 mM CaCl_2_ 1 mM MgSO_4_ for 48 h at 37°C (representative of *n* = 3 independent experiments).

Fourier transform infra-red analysis (Figure 11) reveals bands at 3,400–3180, 2,914, 1,059, and 938 cm^–1^, which in the literature has been ascribed to NH or OH function groups, aldehydic C-H stretching Amide I bands, aliphatic C-N, and D-H deformation (Kitching et al., 2015).

**FIGURE 12:**
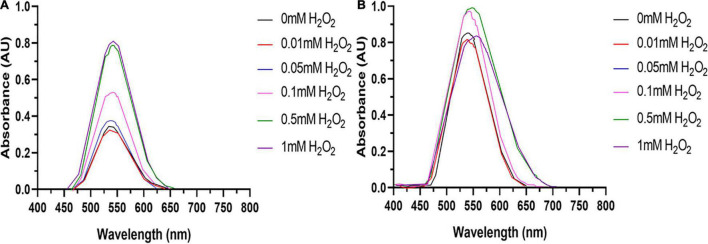
**(A)** BAECs and **(B)** BASMCs pretreated with various concentrations of H_2_O_2_ for 24 h and then exposed to 1.5 mM HAuCl_4_ in 10 mM PBS with 1 mM CaCl_2_ and 1 mM MgSO_4_ ions supplemented with 1% v/v FBS (representative of *n* = 3 independent experiments).

**FIGURE 13:**
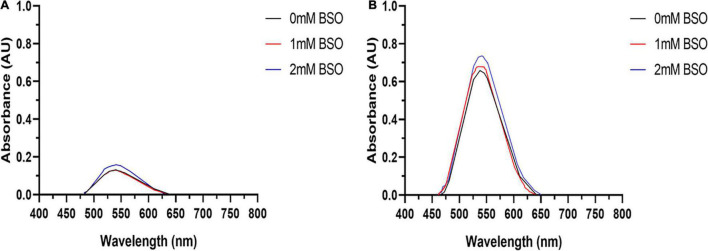
U.V. visible of AuNPs isolated from **(A)** BAECs and **(B)** BASMCs pretreated with 1 and 2 mM BSO for 15 h and then exposed to 1.5 mM HAuCl_4_ in 10 mM PBS with 1 mM CaCl_2_ and 1 mM MgSO_4_ ions supplemented with 1% v/v FBS (representative of *n* = 3 experiments).

Tumor cell lines are hypothesized to have a greater capacity for AuNPs synthesis due to their potentially higher concentration of intracellular ROS (Wang et al., 2013). Therefore, to determine if excess ROS production increase AuNPs biosynthesis, BAECs and BASMCs were pretreated with various concentrations of H_2_O_2_ for 24 h to generate excess intracellular ROS before Au^3+^ exposure. Figure 12 shows that for BAECs and BASMCs, low-level exposure to H_2_O_2_ (1 mM) significantly increases the abundance of AuNPs as determined by the SPR peak height. BASMC mediated AuNPs synthesis decreases upon pretreatment with 1 mM H_2_O_2_. At this concentration, H_2_O_2_ may be inducing apoptosis in BASMC cell lines. As GSH is an important regulator of oxidative stress in mammalian cells, BAECs and BASMCs were pretreated with BSO, which lowers cellular GSH levels *via* irreversible inhibition of the enzyme γ-glutamylcysteine synthetase (Takahashi et al., 2010). However, pretreatment of BAECs or BASMCS with BSO did not significantly affect AuNPs biosynthesis (Figure 13).

## Discussion

This is the first study to demonstrate that vascular cell lines facilitate AuNPs synthesis upon exposure to HAuCl_4_ under nutrient-limited conditions. The choice of buffer influences AuNPs synthesis due to the high reactivity of Au^3+^ ions in the solution. Most studies utilize PBS to avoid potential abiotic reduction or complexation of Au^3+^ ions by media components, which interferes with Au^3+^ bioreduction and biogenesis of AuNPs. Cell lines exposed to Au^3+^ in the DMEM medium failed to produce any AuNPs. DMEM contains potential Au^3+^ complexing agents, which include amino acids such as cysteine (Jin et al., 2012), histidine (Cuadrado et al., 2000), tryptophan (Koleva et al., 2007), and vitamins such as niacinamide (Al-Saif and Refat, 2012) and pyridoxal (Alibrahim et al., 2018). Complexation of Au^3+^ ions lowers their availability for bioreduction and AuNPs synthesis. At high HAuCl_4_ concentration, reducing agents such as glucose reduce and precipitate Au^3+^ abiotically (Supplementary Figure 1).

AuNPs biosynthesis is influenced by synthesis buffer formulation, such as the concentration of FBS and the presence of Ca^2+^ and Mg^2+^ ions. These common constituents of cell culture media are required for the regulation of cellular metabolism, intracellular signaling, and cell adhesion (Takeichi and Okada, 1972; Ko et al., 2001; Sotomayor and Schulten, 2008; Pirkmajer and Chibalin, 2011; Jahnen-Dechent and Ketteler, 2012). Serum deprivation alters cell metabolism (Golpour et al., 2014) and can trigger apoptosis (Higuchi, 2006). FBS also contains anti-oxidants (Tangtrongsup and Kisiday, 2017) and metal-binding proteins (Tkaczyk et al., 2010). Therefore, FBS is usually included at a low concentration in studies examining oxidative stress. For example, Takahashi et al. used 2% v/v FBS when examining the effect of BSO on methylglyoxal-induced apoptosis of BAECs (Takahashi et al., 2010). In this study, at an FBS concentration below 1% v/v, serum deprivation inhibits Au^3+^ bioreduction possibly through alterations of cell metabolism. However, when the concentration of FBS in the synthesis buffer is above 1% v/v, metal-binding proteins derived from FBS complex Au^3+^ ions in solution may also lower intracellular ROS concentration. These results suggest that Au^3+^ reduction is facilitated at least in part by cellular metabolism, meaning that viable and active cells are required for AuNPs synthesis.

Ca^2+^ and Mg^2+^ ions are important allosteric regulators of adhesion proteins, which facilitate cell attachment to the plate surface (Sotomayor and Schulten, 2008; Nunes et al., 2018). An increase in AuNPs production upon supplementing the synthesis buffer with these ions may be due to improved cellular adherence, which leads to increased viable cell abundance. Interestingly, HAuCl_4_ appeared to improve cell attachment in the absence of Ca^2+^ and Mg^2+^ ions. However, the mechanism by which HAuCl_4_ facilitates cell attachment remains unclear.

AuNPs produced by BAECs and BASMCs shows a strong SPR peak at 530 nm, which is characteristic of AuNPs of ∼30 nm in diameter (Agarwal et al., 2015). TEM imaging of AuNPs produced by BAECs and BASMCS show they are spherical with mean diameters of ∼23 nm. The mean diameters of the biosynthesized AuNPs populations are similar to AuNPs synthesized by other mammalian cell lines reported in the literature. For example, the secretome of an MCF7 cell line produced AuNPs with a mean diameter of 30.4 ± 0.6 nm (Soliman et al., 2020). The size distribution of the biogenic AuNPs is broad for both BAEC and BASMC, with a range between 5 and 60 nm. This wide size distribution is typical for AuNPs produced by microbial hosts (Suresh et al., 2011). However, this issue may be cell type-dependent. HepG2 produces small, monodisperse AuNPs with a mean diameter of 2 nm and a size range between 2 and 3 nm. The authors suggested that the higher levels of ROS in cancer cells increase AuNPs biosynthesis (Wang et al., 2013). This may also result in a small, monodisperse AuNPs population due to an increased Au^3+^ reduction rate. However, it may be more likely that there is a higher protein concentration due to the great abundance of cells in tumor cell cultures, as tumor cells are typically not subject to contact inhibition of their growth. As a result, biogenic AuNPs are smaller due to the higher protein: Au^3+^ precursor ratio (Das et al., 2012a).

Dynamic light scattering (DLS) analysis revealed that AuNPs have a larger hydrodynamic size than the core particle size, as determined by TEM analysis (∼74 nm). This is not unexpected, as biogenic AuNPs are capped by proteins derived from the cells, which increases the particle hydrodynamic size. FTIR suggests that proteins derived from the cell cap the biosynthesized AuNPs. However, FTIR is a broad characterization technique, and would not be able to show the differences in proteins that make up the capping layer. It may also be possible that albumin derived from FBS may also be involved in the capping for the AuNPs (Yamamoto et al., 2018).

X-ray diffraction analysis demonstrates that AuNPs produced by BASMC have an Au (111), Au (220), and Au (311) plane, while AuNPs produced by BAECs have an Au (111), Au (220) but not the Au (311) plane. SAED analysis confirms that the AuNPs produced by BASMCs have the Au (111), Au (220), and Au (311) plane and AuNPs produced by BAECs possess the Au (111), Au (220), and not Au (311) plane. Previous XRD analysis on AuNPs produced by HEK-293 and HeLa cells located in the intracellular environment also showed Au (111), Au (200), Au (220) planes, and not the Au (311) plane. As with this study, the Au (111) plane is the most dominant in the XRD analysis of the AuNPs produced by HEK-293 and HeLa (Anshup et al., 2005). Interestingly, XRD analysis of AuNPs produced by an MCF7 secretome showed the AuNPs only had the Au (111) plane (Soliman et al., 2020). These results suggest that cell type may have a subtle effect on AuNPs crystallization, possibly due to differences in Au^3+^ reduction rate.

HR-TEM analysis shows that the d spacing for the AuNPs is 0.23 ± 0.1 nm for BAEC and 0.23 ± 0.2 nm for BASMC, which was used along with the data for the Au (111) plane to calculate crystal size. As Au crystals have a cubic structure, the Au (111) plane is sufficient. The crystal size is much smaller than the particle size, and the wide XRD peaks demonstrate that the particles are semicrystalline, possibly due to the temperature of their synthesis.

Oxidative stress on vascular cell lines such as endothelial cells and smooth muscle cells induces a variety of pathophysiological effects including proliferation and migration, activation of inflammatory signals, and formation of peroxynitrite (ONOO^–^) and is involved in the development of cardiovascular diseases such as atherosclerosis (Burtenshaw et al., 2019). Therefore, vascular cells possess mechanisms to regulate their intracellular redox state to maintain cellular homeostasis (Burtenshaw et al., 2019).

Bovine aortic smooth muscle cells appear to produce more AuNPs than BAECS. However, BASMCs have an apparent lower resistance to H_2_O_2_ pretreatment compared to BAECs. Vascular endothelial cells such as BAECs are in constant contact with the bloodstream, and therefore encounter high shear forces, which induces oxidative stress (Coyle and Kader, 2007). Therefore, these cells may innately possess a higher capacity to respond to ROS generation compared to BASMC, resulting in a higher intracellular concentration of ROS in BASMCs. Therefore, BASMCs cells may produce a higher intracellular concentration of ROS post H_2_O_2_ stimulation and/or Au^3+^ exposure, which leads to a significantly higher Au^3+^ reduction. H_2_O_2_ has been demonstrated to increase the activity of NOS and NOX enzymes, resulting in an increase of O_2_^–^ production, leading to oxidative stress in PAECs (Coyle and Kader, 2007). This suggests that oxidative stress primes the cell to reduce Au^3+^ possibly by increasing anti-oxidant enzyme expression.

Glutathione is an important antioxidant in the cell and is synthesized in a two-step process. Initially, γ-glutamylcysteine synthetase (γ-GCS) combines glutamate and cysteine to form γ-glutamyl cysteine (γ-EC). Glutathione synthetase (GS) catalyzes the condensation reaction between the γ-EC and glycine (Gly) to formed reduced GSH. To maintain healthy redox homeostasis, the balance between GSH and oxidized glutathione (GSSG) is strictly maintained. Upon the introduction of oxidative stress by ROS, glutathione peroxidases (GPX) utilize GSH to neutralize the ROS, which in turn converts GSH to GSSG (Liu et al., 2015). Vascular cells such as HDMEC increased GPX activity upon treatment with 0.5 mM H_2_O_2_ (Tsaryk et al., 2007). GSH has been shown to produce AuNPs *via* reduction of Au^3+^ and capping nanoparticles growth (Mao et al., 2011). BSO pretreatment lowers the cellular abundance of GSH *via* the inhibition of γ-glutamylcysteine synthetase. A lower concentration of GSH renders BAECs unable to respond to oxidative stress induced by methylglyoxal and eventually leads to apoptosis (Takahashi et al., 2010). Pretreatment with BSO did not affect AuNPs biosynthesis by BAECs or BASMC, which suggests that GSH may have no role in AuNPs biosynthesis in BAECs or BASMCs or there is sufficient redundancy in antioxidant defense to sufficiently reduce Au^3+^.

## Data Availability Statement

The raw data supporting the conclusions of this article will be made available by the authors, without undue reservation.

## Author Contributions

MK performed most of the experiments in this manuscript. SI performed XRD analysis of AuNPs. YG analyzed AuNPs *via* HR-TEM. MR advised on the interpretation of FTIR analysis of AuNPs. MK drafted the manuscript, which was revised by EM and PC. All authors contributed to the article and approved the submitted version.

## Conflict of Interest

The authors declare that the research was conducted in the absence of any commercial or financial relationships that could be construed as a potential conflict of interest.

## Publisher’s Note

All claims expressed in this article are solely those of the authors and do not necessarily represent those of their affiliated organizations, or those of the publisher, the editors and the reviewers. Any product that may be evaluated in this article, or claim that may be made by its manufacturer, is not guaranteed or endorsed by the publisher.

## Supplementary Material

Supplementary Figure 1 UV visible spectra of the AuNP isolated from BAECs which were exposed to various concentrations of HAuCl_4_ in **(A)** DMEM (no phenol red) and **(B)** 1 × PBS experiments without BAECs (abiotic) were also performed for **(C)** DMEM and **(D)** 1 × PBS as before hours at 37°C and 5% CO_2_ under static conditions.

## Abbreviations

AuNPs: gold nanoparticles
BAEC: bovine aortic endothelial cell
BASMC: bovine aortic smooth muscle cell
BSO: buthionine sulphoximine
DLS: dynamic light scattering
DMEM: Dulbecco’s Modified Eagle Medium
FBS: fetal bovine serum
FTIR: Fourier transform infra-red
γ-GCS: γ-glutamylcysteine
γ-EC: gamma-glutamylcysteine
GPX: glutathione peroxidase
GSH: glutathione
GSSG: oxidized glutathione
HCAEC: human coronary artery endothelial cells
HDMEC: Human dermal microvascular endothelial cells
HO-1: heme-oxygenase-1
HVSMC: human vascular smooth muscle cells
MnSOD: manganese superoxide dismutase
NADH: nicotinamide adenine dinucleotide
NIH: National Institute of Health
NOS: nitric oxide synthase
NOX: NADH oxidase
PAEC: porcine aortic endothelial cells
PBS: phosphate buffer saline
ROS: reactive oxygen species
RPMI: Roswell Park Memorial Institute Medium
SAED: selected area electron diffraction
SOD: superoxide dismutase
SPR: Surface Plasmon Resonance
RNS: reactive nitrogen species
TEM: transmission electron microscopy
UV: ultra-violet
XRD: x-ray diffraction.
