# Supplementary figures and images for: Biosynthesis of Gold Nanoparticles by Vascular Cells in vitro
Source: Front Microbiol. 2022 Apr 11;13:813511. doi: 10.3389/fmicb.2022.813511 (PMC9036376; doi:10.3389/fmicb.2022.813511)

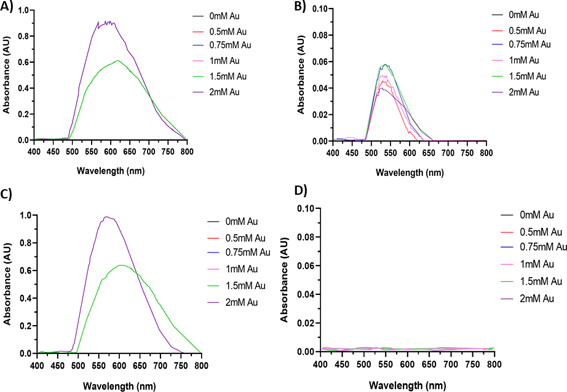

Supplement: Supplementary Figure 1 — UV visible spectra of the AuNP isolated from BAECs which were exposed to various concentrations of HAuCl4 in (A) DMEM (no phenol red) and (B) 1 × PBS experiments without BAECs (abiotic) were also performed for (C) DMEM and (D) 1 × PBS as before hours at 37°C and 5% CO2 under static conditions. [file Image_1.TIF]
